# Healthcare-associated exposure to Borna disease virus 1 (BoDV-1)

**DOI:** 10.1186/s12995-022-00353-3

**Published:** 2022-06-09

**Authors:** Judith Reinmiedl, Heiko Schulz, Viktoria C. Ruf, Moritz R. Hernandez Petzsche, Jürgen Rissland, Dennis Tappe

**Affiliations:** 1 Occupational Medical Services & Occupational Health Management, Ludwig-Maximilians-University Munich, Munich, Germany; 2 Institute of Pathology, Ludwig-Maximilians University Munich, Munich, Germany; 3 Center for Neuropathology and Prion Research, Ludwig-Maximilians-University Munich, Munich, Germany; 4 Department of Diagnostic and Interventional Neuroradiology, School of Medicine, Klinikum rechts der Isar, Technical University of Munich, Munich, Germany; 5 Institute for Virology, Universität des Saarlandes, Homburg/Saar, Germany; 6 Bernhard Nocht Institute for Tropical Medicine, Bernhard-Nocht-Str. 74, 20359 Hamburg, Germany

**Keywords:** Postexposure prophylaxis, Bornavirus, Serology, Imaging, Favipiravir

## Abstract

The Borna disease virus 1 (BoDV-1) causes severe and often fatal encephalitis in humans. The virus is endemic in parts of Germany, Liechtenstein, Switzerland and Austria. As an increasing number of human BoDV-1 encephalitis cases is being diagnosed, the chance for healthcare professionals to come into contact with infected tissues and bodily fluids from patients with known acute bornavirus encephalitis is also increasing. Therefore, risk assessments are needed. Based on three different incidences of possible exposure to BoDV-1 including an autopsy knife injury, a needlestick injury, and a spill accident with cerebrospinal fluid from patients with acute BoDV-1 encephalitis, we perform risk assessments and review published data. BoDV-1 infection status of the index patient’s tissues and bodily fluids to which contact had occurred should be determined. There is only scarce evidence for possible postexposure prophylaxis, serology, and imaging in healthcare professionals who possibly came into contact with the virus. Despite decade-long laboratory work with BoDV-1, not a single clinically apparent laboratory infection has been published. Given the increasing number of severe or fatal BoDV-1 encephalitis cases, there is a growing need for efficacy-tested, potent antiviral therapeutics against BoDV-1 in humans, both in clinically ill patients and possibly as postexposure prophylaxis in healthcare professionals.

## Background

The Borna disease virus 1 (BoDV-1; species *Mammalian orthobornavirus* 1) is one of the two known zoonotic members of the *Bornaviridae* family. BoDV-1 causes animal Borna disease (BD), a non-purulent meningomyeloencephalitis of mainly horses and sheep in virus-endemic regions of Germany, Liechtenstein, Switzerland and Austria [1]. The insectivorous bicoloured white-toothed shrew (*Crocidura leucodon*) is a natural reservoir [1]. The zoonotic potential of BoDV-1 was shown in a series of transplant-related BoDV-1 human encephalitis cases [2] and in one fatal sporadic unrelated case [3] in Germany in 2018. Since then, nearly 40 sporadic cases of human BoDV-1 encephalitis have been reported to health authorities or have been published in Germany, some acute and some retrospectively detected. In 2020, the direct detection of bornavirus infections in humans was made legally notifiable by the German Infection Protection Act (Infektionsschutzgesetz, IfSG). BoDV-1 may be responsible for a considerable proportion of fatal encephalitis cases of previously unknown origin [4–6] in all areas endemic for animal BD in Europe. Human bornavirus encephalitis is believed to have a high case-fatality rate [4]. As an increasing number of severe or fatal BoDV-1 encephalitis cases are diagnosed – likely owing to increased awareness – the chance of healthcare professionals to come into contact with infected tissues and bodily fluids of patients with known bornavirus encephalitis will also increase.

We here describe and review three incidences of possible exposure to BoDV-1 of medical personnel after accidental contact to tissues and bodily fluids from patients with acute BoDV-1 encephalitis. We performed risk assessments and discuss possible postexposure procedures.

## Main text

### Case 1 – knife injury during autopsy

#### Index patient

A 78-year-old female from a rural region of Bavaria had developed encephalitis in 2020 and died after a disease course of approximately 6 weeks. Diagnosis was established by immunohistochemistry (IHC; [2]) and quantitative real-time PCR (qPCR; [2]). According to proposed case definition criteria [4], the patient is a confirmed case of BoDV-1 encephalitis.

#### Contact patient, incident and procedure

**Fig. 1:**
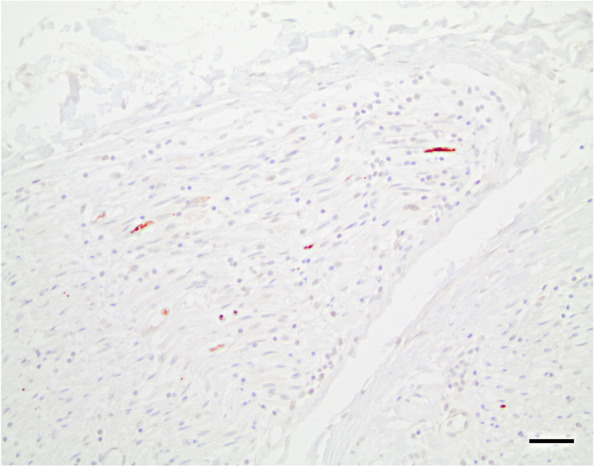
BoDV-1 detection in a peripheral nerve. Positive immunostaining for BoDV-1 phosphoprotein in peripheral nerve surrounding hepatoduodenal lymph nodes – index patient, scenario 1. Scale bar: 50 μm

After autopsy of the index patient, a member of the pathology team accidently touched the blade of a used knife which resulted in a cut injury of the finger penetrating through the surgical gloves. Since the pathology and the neuropathology team used different instruments, the knife had been used to cut non-neural tissue (e.g. liver, kidneys, spleen, soft tissue). After the incident the wound was bleeding without application of pressure and wound disinfection was performed. All available formalin-fixed paraffin-embedded (FFPE) non-neural tissue samples were tested for BoDV-1 using IHC, in situ-hybridization (ISH; [7]) and qPCR. Of 19 tested samples including heart, lungs, kidney, liver, spleen, pancreas, thyroid gland, adrenal glands, bone marrow, small intestine, appendix, and mammary glands, only one sample with soft tissue and lymph nodes was weakly positive by qPCR. Positive IHC staining confirmed the presence of BoDV-1 in a peripheral nerve (Fig. 1) surrounding hepatoduodenal lymph nodes. Results for bornavirus serology were negative in the contact patient directly after the incident, and three and twelve months later, based on a screening assay (indirect immunofluorescence assay with a persistently BoDV-1 infected cell line [4]) and confirmation assay (line blot with recombinant BoDV-1 P antigen [4]). Nine months after exposure, cranial magnetic resonance imaging (cMRI) was performed in the contact patient. No pathologic changes were observed.

### Case 2 – Needlestick injury in an intensive care unit

#### Index patient

A 25-year-old female from a rural region of Bavaria had developed encephalitis of unknown etiology and died in 2005 after a 2-month course of illness. In 2019, the former treating intensive care unit physician (“contact patient” in this scenario, below) has informed the Zoonotic Bornavirus Consortium (ZooBoCo; www.zooboco.fli.de) of this case after he had learned of severe human BoDV-1 encephalitis cases in Bavaria following a nation-wide awareness campaign for neurologists and neuropathologists [6]. Thereupon, archived FFPE brain tissue of the index patient was analyzed by ISH and qPCR for BoDV-1 in 2020, and the results were positive. According to the case definition criteria [4], the index patient had a confirmed BoDV-1 encephalitis.

#### Contact patient, incident and procedure

The contact patient, a former treating physician of the index patient, remembered having had a needlestick injury in his finger while fixing the index patient’s central venous catheter in 2005. The fixation was performed with a surgical needle. Immediately after the accident, bleeding of the injury site was induced by applying pressure to the finger, followed by wound disinfection. Bornavirus serology was performed in 2020, 15 years after the incident, and was negative in the contact patient.

### Case 3 – skin contamination with patient cerebrospinal fluid

#### Index patient

A 64-year-old male from a rural region of Bavaria had developed encephalitis in 2021 and died after a disease course of several weeks. Diagnosis was established *intra vitam* by antibody detection in serum and CSF, as well as by IHC, ISH and qPCR from brain tissue after autopsy. According to case definition criteria [4], this case is also a confirmed BoDV-1 encephalitis.

#### Contact patient, incident and procedure

The contact patient, a laboratory physician, had pipetted CSF of the index patient in a safety cabinet for virological analyses. During this procedure he had spilled several microliters of the fluid over his gloved hand. When removing the glove, the fluid spread on the skin of his hands. Immediately after the incident, hands were washed and disinfected. qPCR testing of the index patient’s CSF was negative. No bornavirus serology was performed in the contact patient.

### Incubation time and transmission routes of BoDV-1

The incubation period in humans is unknown, but ranges from a few weeks to months in naturally infected animals [8, 9]. The transmission routes leading to human infection remain to be elucidated as well, but injured skin and mucosa may probably serve as portals of entry after contact to the virus. In horses, infection with BoDV-1 is assumed to occur via the olfactory nerve after contact to soil contaminated with virus from its natural reservoir, the bicolored shrew [10]. There is no indication of natural human-to-human transmission [5], however, as exemplified by the solid organ-transplant cluster [2], iatrogenic transmissions may occur. In health care settings involving penetrating injuries with virus-infected tissues or CSF as described here in our report, transmissions might also be possible. BoDV-1 is neurotropic. Virus detection outside the central nervous system (CNS) has been described for peripheral nerves [2, 7, 11], which we here also illustrate in case 1. Therefore, the knife cut accident must be seen as a potential exposure. The highest risk of healthcare-associated infection would likely be a penetrating knife injury with unfixed brain or other CNS material. According to the current knowledge, BoDV-1 does not cause a detectable viremia, and spread of the virus in infected hosts occurs via neuronal axons and synapses [10], or by infected transplants [2]. Thus, contact to blood of an infected patient, even by a penetrating injury as demonstrated in case 2, is unlikely to lead to an infection. BoDV-1 is also strongly cell-bound, and the CSF of patients with BoDV-1 encephalitis is often negative when tested by PCR [4, 5], rendering exposure to CSF as described in case 3 also less problematic. None of the three contact patients did develop any neurological symptoms.

### Prevention

In clinical medicine, during lumbar puncture of patients with BoDV-1 encephalitis, double surgical gloves and safety goggles should be worn. In virology, microbiology and laboratory medicine, patient CNS tissues and CSF should be handled with double gloves in a safety cabinet. Safety goggles should be worn. At autopsy, safety goggles or visors, masks (rated FFP2, KN95 or N95) and safety gloves (e.g. kevlar gloves) should be worn. As the highest infectious risk is considered to emerge from unfixed brain tissue, it is recommended to perform body autopsy before brain autopsy. Dissection of the skull should be performed with an easy to decontaminate stainless steel handsaw (instead of an oscillating saw) to avoid formation of airborne particles. Adequately formalin fixed tissue can be regarded as non-infectious.

### Procedures following possible exposure

**Fig. 2:**
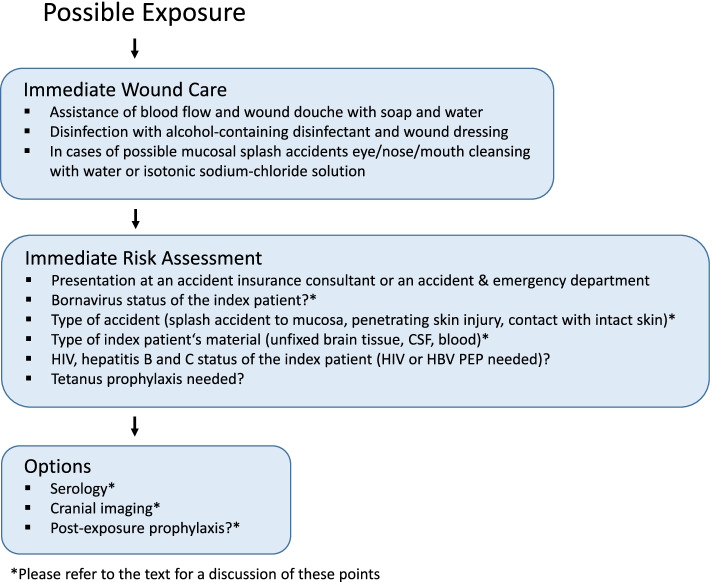
Schematic drawing of postexposure procedures discussed in the text. After a possible exposure, immediate wound care should be performed, followed by gathering information about the bornavirus status of the index patient, the type of accident, and to which possibly virus contaminated material the contact patient was exposed. Further options, such as serology, imaging and the question of a possible postexposure prophylaxis may be considered depending on the individual scenario

As with other enveloped viruses, assistance of blood flow, wound douche with water followed by disinfection with alcohol-containing disinfectants, wound closure and, in cases of possible mucosal splash accidents, eye/nose/mouth cleansing with water or isotonic sodium-chloride solution is strongly advisable. For the risk assessment, the status of infectivity (by direct pathogen detection) of the index patient and his bodily fluids and tissues, as illustrated in the three cases, are important. Moreover, serological testing of the exposed person as basic status is advisable. Presentation at an accident insurance consultant or an accident and emergency department, documentation of the incidence as well as a check of the vaccination status (e.g. hepatitis B or tetanus) alongside with conventional diagnostics (e.g. HIV, HBV and HCV) belong to the principal actions after an occupational exposure (Fig. 2).

### The role of serological testing

Rapid *intra vitam* diagnosis of probable bornavirus encephalitis cases can be achieved by antibody detection in serum and CSF, based on a recently published testing scheme [4]. The detection of bornavirus-reactive antibodies in serum is more sensitive than serology from CSF [4]. The time to seroconversion, however, is variable, and some BoDV-1 encephalitis patients are seropositive at the time of hospitalization, whereas others show detectable antibodies only shortly before death [2, 4, 5].

Therefore, for healthcare-associated exposure, it is unclear whether a seroconversion would develop before the onset of encephalitis. We decided to offer serologic testing to the patients nonetheless, in one patient (case 1; with the most risky situation) in a follow-up scheme.

### The role of imaging studies

**Fig. 3:**
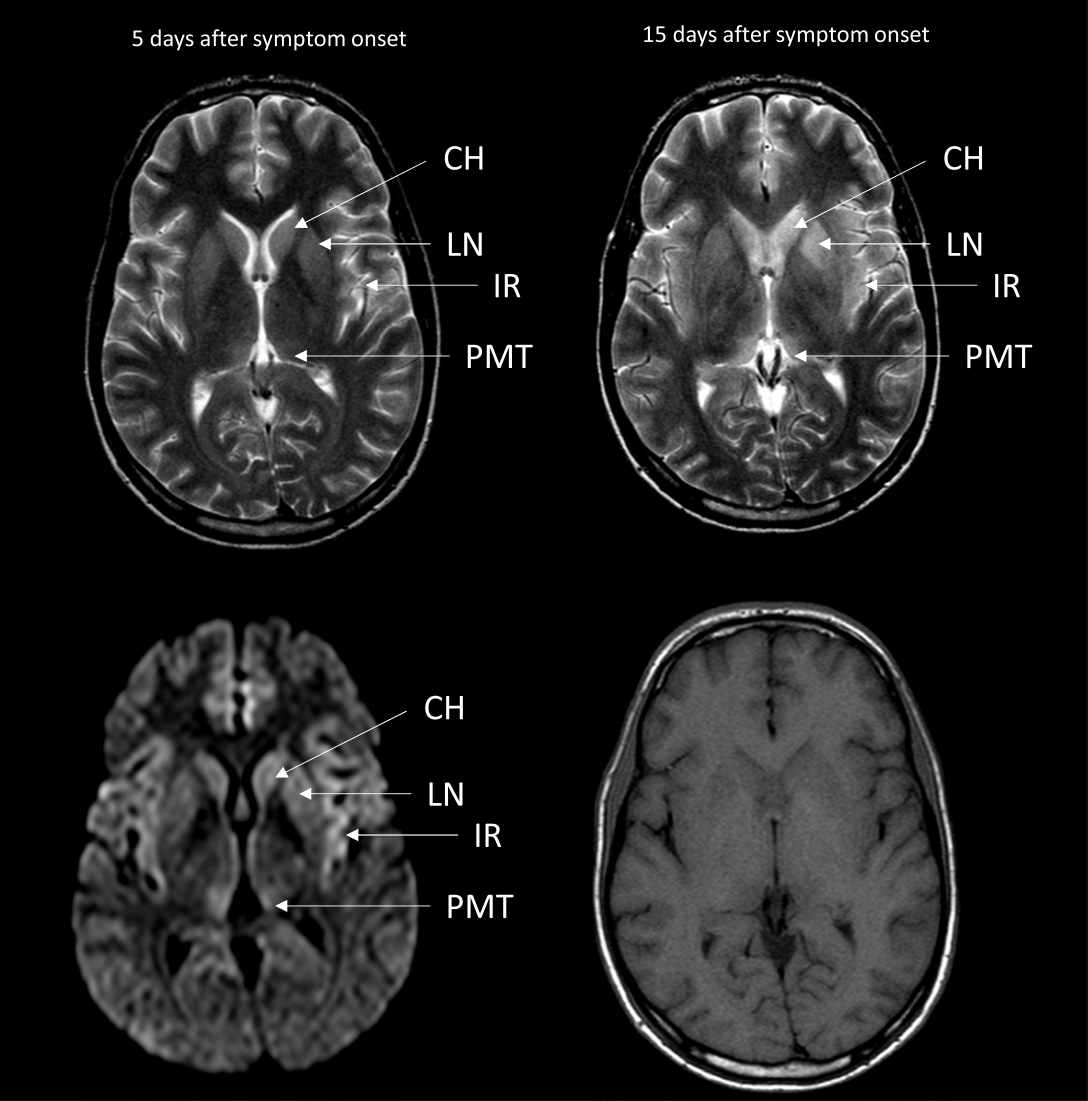
Typical cranial magnetic resonance imaging of early BoDV-1 encephalitis. Left column: T2 weighted imaging (top left) shows discrete, nearly symmetric hyperintensities of the insular ribbon (IR), the posteromedial thalamus (PMT), the caudate head (CH) and the lentiform nucleus (LN). These changes are more easily appreciated on diffusion-weighted imaging (DWI, bottom left), where diffusion restriction in these brain areas is visualized as a hyperintense signal. Right column: T2 weighted imaging (top right) shows progressive T2 hyperintensities of the affected areas with associated parenchymal swelling, somewhat more pronounced on the patient’s left side. No T1 hyperintense signal changes, which are characteristic of late-stage BoDV-1 encephalitis, were present at this time point (bottom right)

A recent study was able to determine a highly characteristic pattern of MRI findings in patients with manifest BoDV-1 encephalitis [12]. Early stage findings include nearly symmetric T2 hyperintensity and diffusion restriction affecting the posteromedial thalamus, the head of the caudate nucleus, the insular cortex, and the hippocampus (Fig. 3). In the later stages of the disease, T1 hyperintensities are observed in the basal ganglia. Such cMRI findings should raise the suspicion for a bornavirus encephalitis and prompt the implementation of safety recommendations for healthcare professionals as described above.

Owing to the scarcity of studies on the subject, the role of cMRI in pre-symptomatic bornavirus encephalitis remains unclear. Pathological findings in cMRI have been shown to precede serious symptoms in prion-induced variant Creutzfeldt–Jakob disease of the brain after occupational exposure [13]. However, due to the different pathogenesis of these two illnesses, it is unclear whether cMRI is suitable for screening after BoDV-1 exposure. As was performed in case 1 of this series, cMRI may be considered after 6 to 12 months post exposure. cMRI should be performed immediately in the case of new-onset neurological symptoms after virus exposure.

### Potential postexposure prophylaxis

There is neither a medical postexposure prophylaxis (PEP), nor treatment available for human bornavirus encephalitis. Ribavirin (1-β-D-ribofuranosyl-1,2,4-triazol-3-carboxamide, a guanosine analog) and favipiravir (T-705; 6-fluoro-3-hydroxypyrazine-2-carboxamide, a synthetic guanidine nucleobase) show promising in vitro efficacy against BoDV-1. Both drugs inhibit RNA virus replication by targeting viral polymerases and thus suppress viral RNA levels, but favipiravir acts more efficiently [14]. In vivo animal testing results have not been published so far for favipiravir and BoDV-1. Published results for favipiravir and rabies virus, which is also a member of the *Mononegavirales,* showed that the drug efficiently suppressed rabies virus replication at the inoculation site and the subsequent replication in the CNS [15]. There is currently no data about the CSF penetration of favipiravir in humans. Ribavirin, however, has been shown to penetrate into the CSF in humans [16]. In animal models with BoDV-1, ribavirin administered intrathecally has shown clinical improvement [17]. Both drugs have been used in a few BoDV-1 encephalitis cases, but very late in the course of disease, and have shown no effect so far (personal communications).

Thus two potentially therapeutic drugs are available, yet in vivo data of animal treatment for BoDV-1 or human treatment for other viruses are scarce. Whether the drugs, when used as PEP, are potent to prevent the development of encephalitis after BoDV-1 exposure remains unclear. If an administration should be considered at all, then most likely in the highest risk setting, such as in penetrating skin injuries or mucosal splash accidents with index patient CNS tissues during autopsy.

## Conclusions

In the future, more exposure incidents of healthcare professionals to BoDV-1 are to be expected and we aimed with this analysis to highlight prevention and possible postexposure procedures. However, routes of transmission, incubation time, likelihood of infection, time to seroconversion and efficacy of PEP in humans following possible exposure to BoDV-1 are so far best guesses from scarce scientific evidence. The incubation time might be dependent on the portal of entry and the amount of virus transmitted.

Despite decade-long worldwide experimental laboratory work with BoDV-1, a pathogen still to be handled under biocontainment level 2 in Germany [18], not a single clinically apparent laboratory infection has been published so far. However, given the increasing number of severe or fatal BoDV-1 encephalitis cases, there is an urgent need for efficacy-tested, potent antiviral therapeutics against BoDV-1 in humans.

## Data Availability

All data and material, when applicable, are available upon request.

## Abbreviations

BD: Borna disease
BoDV-1: Borna disease virus 1
cMRI: Cranial magnetic resonance imaging
CSF: Cerebrospinal fluid
CNS: Central nervous system
FFPE: Formalin-fixed paraffin-embedded
HIV: Human immunodeficiency virus
HBV: Hepatitis B virus
HCV: Hepatitis C virus
IHC: Immunohistochemistry
ISH: In situ-hybridization
PEP: Postexposure prophylaxis
RNA: Ribonucleic acid
qPCR: Quantitative real-time polymerase chain reaction
